# Plant Clinics for Improved Plant Health Systems—Malawian Plant Doctor Insights

**DOI:** 10.1002/pei3.70056

**Published:** 2025-05-09

**Authors:** Maureen Mildred Banda, Mariam A. T. J. Kadzamira, Noah Phiri

**Affiliations:** ^1^ Citizen Pillar Lilongwe Malawi; ^2^ CABI UK Centre Egham UK; ^3^ Formally With CABI Zambia Centre Lusaka Zambia

## Abstract

This study provides qualitative insights on the factors affecting the implementation of the plant clinic approach in Malawi from the viewpoint of plant doctors. Findings show that plant doctors perceive that the main benefit of plant clinics in Malawi has been the strengthening of local agricultural extension service systems in plant health disease diagnosis and pest management. The full potential of the plant clinic approach is, however, perceived as not being fully reached due to various challenges that include but are not limited to the scarcity of trained plant doctors coupled with the lack of sufficient resources to improve plant doctor mobility and to adequately access and use digital plant health resources. Some of these are being overcome through the embedding of the approach in national agricultural extension service systems and strategies, and integration with local plant health activities. For sustained implementation of plant clinics in Malawi, there is a need for continued policy, technical, and financial resources to support plant doctors to better utilize their knowledge to reach more farmers and to leverage digital technology to continuously broaden their plant health capacity through use of and access to mobile technology, digital plant health tools, and virtual plant health expert networks.

## 1 Introduction

Globally, plant pests and diseases present a major obstacle to sustainable agricultural productivity (Vurro et al. 2010; Spence et al. 2020). Left unchecked, pests and diseases can result in significant losses in the quality and quantity of agricultural produce (Oerke 2006; Savary et al. 2012; Sharma et al. 2017). In the past two decades, Malawian agriculture has been affected by climatic vagaries (Saka et al. 2012), and it has witnessed increased infestation of plant pests and diseases (Chimpokosera‐Mseu 2023) resulting in increased food insecurity. In order to sustain agricultural production, various stakeholders have been working with farmers in the country to employ innovative approaches to combat plant pests and diseases.

Plant clinics have been widely established globally as an innovative approach for the provision of demand‐driven plant health advice to farmers (Bentley et al. 2007; Efa et al. 2018; Majuga et al. 2018; Danielsen et al. 2020; Kansiime et al. 2020). Modeled on the concept of human health, plant clinics allow farmers to take their diseased and pest‐infested plants for diagnosis and to receive management recommendations. Evidence exists of the impact of plant clinics on farming communities as well as local plant health systems. For farmers, there is evidence of improved livelihood outcomes as a result of attending plant clinics (Musebe et al. 2018b; Silvestri et al. 2019; Tambo et al. 2020, 2021c, 2021a). In addition, the establishment of plant clinics has increased farmers' ability to identify and manage plant pests and diseases (Rajkumar and Anabel 2018; Silvestri et al. 2019), with clients of plant clinics more likely to adopt Integrated Pest Management (IPM) techniques (Musebe et al. 2018a; Tambo et al. 2021b; Gurmessa and Bundi 2023).

Introduced in 2013 in Malawi, plant clinics aim to help farmers with plant pest and disease diagnosis, with trained plant doctors providing solutions to combat their pest and disease problems (Bentley et al. 2017). Currently, almost all trained plant doctors in Malawi are government extension workers, who are working throughout the country and are responsible for providing agricultural extension services to farmers within their extension planning area (EPA). Initially, plant clinics were designed to take place once a week or once every fortnight at a public place such as a market, a village square, or a trading centre on a designated clinic day. However, this has not been the *modus operandi* for most plant clinics in Malawi due to various demand and supply side factors. Studies that provide robust evidence of the factors confounding the plant clinic approach within the nexus of national plant health systems and agricultural advisory services in Malawi are few (Bett et al. 2018; Bentley et al. 2018) and provide insights solely on effects based on plant clinic and farmer household data, without capturing the perceptions of plant doctors. This study addresses this gap as it aimed to understand the factors confounding the implementation of the plant clinic approach in Malawi, while concurrently unpacking the benefits and opportunities it offers to farming communities and the country's plant health system from the viewpoint of plant doctors. Study findings provide insights for policy makers and plant health practitioners working to improve the functioning of national plant health systems and agricultural advisory services. Lessons drawn from Malawi are applicable to other agro‐dependent countries in the region, who are grappling with similar challenges.

## 2 Methodology

Key informant interviews of plant doctors and of plant health experts in Lilongwe district and across the country, respectively, were conducted to determine their views on the benefits, threats, opportunities, and risks of the plant clinic approach in Malawi. Plant doctors were purposively selected on the basis that they had undergone all or part of the plant doctor training (Toepfer 2023). While plant health experts included plant health and agricultural extension practitioners working either in plant health, agricultural research, or in agricultural extension services in the country, they too had to be experts or practitioners who had either undergone plant doctor training or were seasoned experts who had worked extensively and directly with frontline agricultural extension staff. Sample size increased through snowballing from the first interview, and in total, five key informant interviews (of trained plant doctors) were conducted, mostly via telephone lasting between 30 and 60 min, while one was conducted at a plant clinic location, which included farmers. In addition, insights from the plant doctor key informant interviews were shared with and vetted by five plant health experts, who all support the plant clinic approach nationally. Information collected from plant doctors included background information on the history of the plant clinic (when it was established, frequency of its operation, type of clientele and the engagement with women and youth); type of queries they get from farmers and the type of management solutions they provide; their use of mobile and digital/online resources and tools; and the type of training they had received to become a plant doctor, including the specific modules that they had been trained on. At the plant clinic where farmers were present (a total of three farmers, representing three different farming households, were on site during our visit), we asked them to explain when they started visiting the plant clinic, and if there had been a time when they visited other plant clinics further afield, the type of support they typically received, and the frequency of attending sessions. For all key informants, including the plant health experts, we asked them to tell us the changes that have taken place since plant clinics were established in their area of operation or at the national level, to elaborate on the most significant change and give reasons behind their observations.

A rapid review of the literature was conducted to consolidate the insights from the key informant interviews. Using published literature on plant clinics, we crosschecked findings from three large‐scale studies of the Plantwise Program—specifically CABI (2019), Cantrell (2021) and Danielsen and Cantrell (2023) against insights or themes arising from the key informant interviews (see Table 1). In so doing, the results from the study are discussed with nuances from the literature, thus allowing the drawing of generalized conclusions and recommendations for plant clinics in Malawi, but with lessons for other countries grappling with similar issues.

**TABLE 1:** Malawi plant health clinics—benefits, challenges, opportunities, and threats.

	Description (from focus group discussions)	Published work with similar or contradictory findings
Benefits	− Most trained plant doctors work as government frontline agricultural extension officers. They usually live and work locally with farming communities. − Due to their close proximity, plant doctors are the first point of contact for most farmers. − Plant clinic sessions are often held on market days with farmers from across an entire EPA and beyond attending. − In addition, “mobile” plant clinics are often held during agricultural fairs, farmer field days and other local events, hence broadening their reach.	CABI (2019) Danielsen and Cantrell (2023)
Challenges	− Ratio of farmer to trained plant doctor is high as not all frontline agricultural extension staff have been trained in the plant doctor approach. − Mobility challenges of plant doctors prevents them from visiting farmers' fields as often as they would want to, in order to assess extent of pest damage and infestation. They may only be able to visit farms that are close to the plant clinic venue. − Plant doctors do not always have functional smart phones with which to connect to the internet or access plant health digital tools and services. − Plant doctors sometimes seek support from expert plant doctors (via established online chatrooms or groups) but the response is not always in real time. − Inadequate refresher courses to upgrade plant doctors on emerging pests, diseases and abiotic issues. − Lack of funding to support essential materials for the plant clinics, including lab coats and furniture.	CABI (2019) Cantrell (2021)
Opportunities	− Mobile plant clinics set up during farmer field days, local agricultural shows, and other local events create awareness of the approach and reach a larger number of farmers. − Knowledge from plant clinics is generally transferable, hence farmers who get plant health advice are able to advise fellow farmers who might not attend plant clinics. This has been enhanced in some places by training “lead farmers” to better support their peers with plant health issues. − Plant doctor knowledge is used by plant doctors as part of their day‐to‐day agriculture extension frontline work. − Plant clinic participation has led to increased awareness amongst clientele of lower risk pesticides and of biopesticides. − Availability of digital resources and online chatrooms to support local plant doctors via networks of plant health expertise, thus enabling technical backstopping and access to plant health knowledge and information banks.	CABI (2019) Cantrell (2021) Danielsen and Cantrell (2023)
Threats	− Farmers are unable to access plant clinics or plant doctors on a daily basis or as needed due to scarcity of trained plant doctors. − Plant doctors are faced with limited funding for mobile data and internet to access online plant health resources. − Plant clinic records^a^ are not always up to date or if up to date are in paper format at the plant clinic and not easily transferable to the national Plantwise Online Data Management System (POMS)^b^.	Cantrell (2021) Danielsen and Cantrell (2023)

^a^ Plant clinic records include data that is collected regularly by plant doctors, which captures the number of farmers attending plant clinics, the types of queries they bring, and the type of solutions they are provided with by the plant doctor.

^b^ The Plantwise Online Data Management System (POMS) is a global repository, fed by national systems, of plant clinic data used to estimate attendance, farmer queries, and management solutions provided (Majuga et al. 2018).

## 3 Results and Discussion

Table 1 presents a summary of the findings from the key informant interviews of the benefits, challenges, opportunities, and threats of the plant clinic approach in Malawi. In addition, Table 1 also presents works from the literature that have found similar or, in some cases, contradictory findings—this is not restricted to work on Malawi but plant clinics globally. The main benefit of the approach has been the strengthening of local agricultural extension service systems in plant disease diagnosis and pest management. This is because most of those trained as plant doctors are frontline public extension staff who are stationed across the country in various EPAs. This is also often the case in other countries where plant clinics have been implemented (Toepfer et al. 2023). Hence, they typically live within the farming communities where they work, resulting in greater farmer reach. Interviewees, however, felt that the potential has not been fully realized because training has been provided to relatively few frontline extension staff. Plant doctors who were interviewed stated that the scarcity of trained staff, coupled with a lack of sufficient resources to improve mobility for plant doctors to reach more farmers or to enable them to quickly access digital plant health tools, were the main challenges to effective implementation of the plant clinic approach in their area in the present as well as in the foreseeable future. Similar sentiments have also been echoed in other countries where plant clinics have been implemented (CABI 2019; Cantrell 2021).

Despite these challenges, there are many opportunities that can be leveraged to ensure sustained implementation of plant clinics in Malawi. These focus on the utilization and dissemination of the knowledge gained from plant doctors and visits to plant clinics beyond the plant clinic setting. Furthermore, digital technology offers an opportunity for broadening the knowledge that local plant doctors have through the use of digital plant health tools and online networks of plant health experts such as those documented by Jomantas et al. (2024).

## 4 Conclusions

From interviews with plant doctors, we provide insights on the factors affecting the implementation of the plant clinic approach in Malawi. Findings show that plant doctors perceive that they face various challenges which keep them from reaching their full potential; however, the embedding of the approach in national agricultural extension service systems and integration with local plant health activities has contributed to greater farmer reach, resulting in better management of crop pests and diseases. In order to further improve farmer accessibility and for sustainability, there is a need for greater investment in plant health training and capacity strengthening of frontline agricultural extension officers. Progress towards this has already been made not only in Malawi but also in several countries where the approach is in use (Cantrell 2021). In addition, there is also a need to utilize and support community/village plant health champions (FAO 2020) to better enable them to continue cascading their plant health knowledge to fellow farmers and to advocate for the importance of plant health in their local area. This would entail training them in the use of digital plant health tools so that they can reach out to more of their fellow farmers, especially in areas that are not covered by extension services. All these efforts should be coupled with supporting ‘mobile’ plant clinics, which have organically evolved in Malawi and which have the potential to contribute to greater effectiveness and sustainability. Throughout this process, there is a need to continue supporting plant doctors with technical backstopping as and when needed, virtually where in‐person support is not possible, and to support them to have access to plant health knowledge and information banks. Finally, there is a need to ensure continued integration of the plant clinic approach in agricultural sector policy and strategic documents. This is critical as often embedding in national development plans and strategies raises priority and creates an opportunity for public budget commitments.

## Consent

All authors have read the final manuscript and have given consent for it to be published.

## Conflicts of Interest

Co‐authors M.K. and N.P. are and were formerly employed by the CABI, which is funded by the UK Foreign, Commonwealth and Development Office (FCDO), respectively. Co‐author MB has no interests to declare.

## Data Availability

The data that support the findings of this case study are openly available at: https://drive.google.com/drive/folders/1oefuBADnTCnWk21PT8299rP51aUPLpuh?usp=sharing.
